# 10-HDA Suppresses the Growth of Gastric Cancer Cells Through Inducing Apoptosis and G0/G1 Cell Cycle Arrest

**DOI:** 10.3390/nu18162670

**Published:** 2026-08-15

**Authors:** Lei Huang, Tianxing Lin, Shoujie Jiang, Anqi Lin, Yuqi Zhu, Zhenyu Lin, Yan Lin, Songkun Su

**Affiliations:** 1 College of Animal Sciences, Fujian Agriculture and Forestry University, Fuzhou 350002, China; leiihuang@fafu.edu.cn; 2 College of Bee Science, Fujian Agriculture and Forestry University, Fuzhou 350002, China; tianxinglin0821@fafu.edu.cn (T.L.); 52507057012@fafu.edu.cn (S.J.); 12407044014@fafu.edu.cn (A.L.); 12507044016@fafu.edu.cn (Y.Z.); 3245602021@stu.fafu.edu.cn (Z.L.); 3 College of Life Sciences, Fujian Agriculture and Forestry University, Fuzhou 350002, China

**Keywords:** gastric cancer, 10-hydroxy-2-decenoic acid, royal jelly, apoptosis, cell cycle

## Abstract

**Background/Objectives:** Gastric cancer (GC) remains a major global health challenge, highlighting the need for safe and effective bioactive compounds for prevention and treatment. Although 10-hydroxy-2-decenoic acid (10-HDA)—a distinctive bioactive fatty component of royal jelly—has demonstrated anticancer activity in several malignancies, its efficacy and underlying mechanisms in GC remain poorly understood. In the present study, the anticancer activity of 10-HDA was systematically investigated in human GC MKN-45 cells. **Methods:** MKN-45 cells were treated with 10-HDA across a concentration range of 0.09–1.50 mM. Cell viability, proliferation, and clonogenic growth were evaluated using MTT, scratch wound assay, and colony formation assays. Apoptosis was analyzed by flow cytometry and Western blotting. To explore the underlying molecular mechanisms, transcriptome analysis was conducted, and the results were further validated using flow cytometry, qRT-PCR, and Western blotting. **Results:** Treatment with 10-HDA significantly inhibited cell viability, proliferation, and clonogenic growth in a dose- and time-dependent manner, with IC_50_ values of 1.24 ± 0.15 mM, 0.96 ± 0.08 mM, and 0.42 ± 0.06 mM at 24 h, 48 h, and 72 h, respectively. Flow cytometric analysis revealed that apoptosis was markedly induced, with the apoptotic rate increasing from 21.3% in control cells to 78.1% following treatment with 1.50 mM 10-HDA. This was accompanied by upregulation of BAX, downregulation of Bcl-2, and increased activation of caspase-3. Transcriptomic profiling identified significant alterations in DNA replication- and cell cycle-related pathways. Consistently, experimental validation demonstrated that 10-HDA induced pronounced G0/G1-phase cell cycle arrest; downregulation of CDK2 and cyclin D1; reduced retinoblastoma protein phosphorylation; and upregulation of the cyclin-dependent kinase inhibitor *CDKN1A*. **Conclusions:** These findings demonstrate that 10-HDA suppresses the malignant phenotype of gastric cancer cells by coordinated activation of apoptosis and cell cycle blockade. Although further in vivo studies and pharmacological experiments are required to evaluate its bioavailability and therapeutic potential, this study provides preliminary mechanistic insights into the effects of 10-HDA on gastric cancer cells.

## 1. Introduction

Gastric cancer (GC) is a common malignancy that continues to pose a significant and ongoing challenge to global public health [1]. In 2022, approximately 968,350 new cases (4.9% of all cancer diagnoses) and 659,853 deaths (6.8% of all cancer-related fatalities) were recorded, ranking GC as the fifth most frequently diagnosed cancer and the fourth leading cause of cancer mortality [2]. The global incidence of GC is projected to increase by 62% by 2040 [2]. Most patients with GC are diagnosed with locally advanced disease or distant metastasis at initial diagnosis [3]. Although multimodal treatment strategies, including surgical resection, chemoradiotherapy, and molecularly targeted agents, have been increasingly applied, clinical outcomes remain suboptimal [4]. Chemotherapy resistance develops in 50–70% of patients, driven by molecular adaptations, and leads to disease relapse in more than 60% of treated individuals within five years [5]. These challenges highlight the urgent necessity for the development of effective pharmacological agents to improve outcomes of patients with GC.

Functional foods, distinguished by their rich content of bioactive components, provide health benefits beyond basic nutrition through their antioxidant, anti-inflammatory, and immunomodulatory properties [6]. Characterized by low toxicity and high biocompatibility, increasing attention has recently been directed toward their application in cancer research [7]. The antitumor activity of functional foods is primarily attributed to diverse bioactive constituents, including polyphenols, flavonoids, carotenoids, polyunsaturated fatty acids, bioactive peptides, and organosulfur compounds. These molecules regulate multiple cellular events, such as reactive oxygen species scavenging, attenuation of chronic inflammatory responses, induction of apoptosis, and suppression of angiogenesis, thereby restricting tumor development through the modulation of diverse signaling networks [8,9,10,11,12,13]. In addition, these bioactive components may synergistically enhance the efficacy of chemotherapy and radiotherapy while alleviating their adverse effects [14,15]. Given their multitarget modulatory properties and favorable safety profile, these bioactive components hold considerable potential in cancer prevention and long-term adjuvant therapy.

Produced by worker bees, royal jelly (RJ) is a yellowish-white secretion with a creamy consistency [16]. As a functional food, RJ is highly valued not only for its nutritional profile and unique flavor but also for its broad spectrum of functional properties, including antimicrobial, anti-inflammatory, and tissue-regenerative activities. These properties have historically supported its extensive use as a dietary supplement in traditional medicine [17,18,19]. 10-hydroxy-2-decenoic acid (10-HDA) is a distinctive medium-chain fatty acid exclusive to RJ and represents one of its important active compounds [20]. Previous investigations have demonstrated that 10-HDA exerts multifaceted pharmacological activities, encompassing anti-inflammatory, antioxidant, immunomodulatory, and anticancer properties [21,22,23,24]. Recent studies have shown that 10-HDA exhibits significant anticancer activities against a variety of cancer cell lines, including those derived from colorectal, lung cancer, hepatoma, and breast cancer, and has been shown to effectively mitigate cisplatin-induced nephrotoxicity [25,26,27,28]. Despite these insights, the specific anticancer potential of 10-HDA against human GC cells remains to be elucidated. Therefore, the primary objective of this study was to evaluate the in vitro anti-proliferative effects of 10-HDA in MKN-45 cells and to characterize its roles in apoptosis and cell cycle arrest, thereby providing preliminary baseline data for its cellular mechanisms.

## 2. Materials and Methods

### 2.1. Cell Culture

MKN-45 human GC cells and normal human gastric cells (GES-1) were obtained from the Cell Bank of the Chinese Academy of Sciences (Shanghai, China). MKN-45 cells were grown in high-glucose Dulbecco’s Modified Eagle Medium (DMEM; Gibco, Carlsbad, CA, USA) supplemented with 10% fetal bovine serum (FBS) (ExCell, Beijing, China) and 1% penicillin–streptomycin (Gibco, Carlsbad, CA, USA), whereas GES-1 cells were maintained in RPMI 1640 (Gibco, Carlsbad, CA, USA) containing 10% FBS and 1% penicillin–streptomycin solution. All cells were cultured at 37 °C in a humidified atmosphere containing 5% CO_2_.

### 2.2. Cell Viability Assay

The viability of cells following exposure to 10-HDA was assessed using the 3-(4,5-dimethylthiazol-2-yl)-2,5-diphenyltetrazolium bromide (MTT) assay, as previously described [29]. Briefly, the cells were seeded at a density of 7.0 × 10^3^ cells/well (MKN-45 cells) or 3.0 × 10^3^ cells/well (GES-1 cells) into 96-well plates. After allowing cells to attach for 24 h, they were exposed to various concentrations of 10-HDA (0.09–1.50 mM) (purity ≥ 98%; MedChemExpress, Monmouth Junction, NJ, USA) for 24, 48, and 72 h. A volume of 10 μL of MTT (5 mg/mL; Solarbio, Beijing, China) solution was added to each well and incubated at 37 °C for 4 h to form purple formazan crystals. To dissolve the formazan crystals, 100 μL of dimethyl sulfoxide (Solarbio, Beijing, China) was added per well, and the plate was agitated in the dark for 10 min. The optical density at 550 nm was subsequently recorded using a microplate reader (Thermo Fisher Scientific, Waltham, MA, USA).

### 2.3. Scratch Wound Assay

The effect of 10-HDA on the scratch closure of MKN-45 cells was evaluated by scratch wound assay, as previously described [30]. MKN-45 cells (5 × 10^5^ cells) were cultured in each well of the culture inserts (Ibidi, Gräfelfing, Germany) to form confluent cell monolayers containing a 500-micrometer-wide gap in the middle. The cell monolayers were incubated with 0.09–1.50 mM 10-HDA or medium alone (control) for 72 h. At each time point (0, 24, 48, and 72 h), three randomly selected gap regions in each well were photographed using a phase-contrast microscope (Olympus, Tokyo, Japan), and subsequently analyzed with Image J software (v1.53k; National Institutes of Health, Bethesda, MD, USA). Gap closure rates were calculated as percentages of gap area reduction at each time point relative to the area at 0 h.

### 2.4. Colony Formation Assay

MKN-45 cells were plated in 6-well plates at a density of 5.0 × 10^2^ cells/well and incubated overnight. The cells were then treated with different concentrations of 10-HDA for 48 h, followed by replacement with 10-HDA-free medium. The cells were then continuously cultured for 15 days, with the medium being changed every 3 days. After removing the medium, the cell colonies were washed with PBS, fixed with 4% paraformaldehyde (Servicebio, Wuhan, China) for 20 min, and stained with 0.1% crystal violet (Beyotime, Shanghai, China). Images were captured and analyzed using ImageJ software (v1.53k).

### 2.5. Cell Apoptosis Assay and Cell Cycle Analysis

A total of 3.0 × 10^5^ MKN-45 cells were seeded in 6-well plates and cultured for 24 h. After 24 or 48 h of 10-HDA exposure, cells were collected and washed with PBS, followed by staining with the Annexin V-FITC/7-AAD Apoptosis Detection Kit (YEASEN, Beijing, China) or the Cell Cycle Staining Kit (Lianke, Hangzhou, China) according to the manufacturer’s instructions. Flow cytometry was performed on a BD Accuri C6 Plus (BD, San Jose, CA, USA) to determine apoptosis rate and cell cycle distribution, and the results were analyzed using FlowJo (v10.8.1; FlowJoLLC, Ashland, OR, USA).

### 2.6. RT-qPCR Assays

Total RNA was isolated using the Eastep^®^ Super Total RNA Extraction Kit (Promega, Madison, WI, USA). cDNA was synthesized with the Hiscript^®^ II Q RT SuperMix for qRT-PCR (+ gDNA wiper) kit (Vazyme, Nanjing, China). Quantitative real-time PCR was performed with 2× ChamQ SYBR Color qPCR Master Mix (Low ROX Premixed) (Vazyme, Nanjing, China), and the gene-specific primers are listed in Table S1.

### 2.7. Transcriptomic Analysis

After quality assessment and quantification of total RNA, cDNA libraries were constructed and sequenced with paired-end reads on the Illumina NovaSeq X Plus platform (Illumina, San Diego, CA, USA). Raw reads were trimmed to remove adapter contamination and low-quality bases, producing high-quality clean reads. Clean reads were then mapped to the human reference genome using a splice-aware alignment tool, and gene-level read counts were quantified. Differentially expressed genes (DEGs) between the 10-HDA-treated and control groups were called based on the thresholds of false discovery rate (FDR) < 0.05 and |log2 (fold change)| ≥ 1. Subsequently, Gene Ontology (GO) and KEGG pathway enrichment analyses were performed on the DEGs, and those with an adjusted *p* < 0.05 were considered significantly enriched.

### 2.8. Western Blotting Assays

A total of 3 × 10^5^ MKN-45 cells were seeded in each well of 6-well plates and cultured for 24 h. Cells were subsequently exposed to different concentrations of 10-HDA or to medium alone (control) for 48 h. MKN-45 cells were lysed with RIPA lysis buffer (EpiZyme, Shanghai, China). Following separation on a 12% SDS-PAGE gel, the proteins were transferred to polyvinylidene fluoride (PVDF) membranes. Membranes were blocked and probed overnight at 4 °C with primary antibodies against BAX, Bcl-2, caspase-3, cleaved caspase-3, CDK2, cyclin D1, p-Rb, Rb, PCNA, and actin (all diluted 1:2000; ProteinTech, Wuhan, China). Following washing, membranes were incubated with the appropriate secondary antibody, HRP Goat Anti-Mouse IgG (H+L) (1:20,000; Abclonal, Wuhan, China) or Goat Anti-Rabbit IgG H&L (HRP) pAb (1:20,000; ProteinTech, Wuhan, China), for 2 h at room temperature. Protein bands were visualized using an enhanced chemiluminescence Substrate Kit (ECL; EpiZyme, Shanghai, China) and imaged with a Tanon-6000+ system (TanonImage; Tanon, Shanghai, China).

### 2.9. Statistical Analysis

All data are expressed as the mean ± standard deviation (SD). For experiments involving multiple dose groups compared to a control group, statistical significance was analyzed using one-way ANOVA followed by Dunnett’s multiple comparisons test. A *p* < 0.05 was considered statistically significant. Statistical analysis and graph generation were performed by Prism 8.0 (GraphPad Software, San Diego, CA, USA).

## 3. Results

### 3.1. 10-HDA Suppresses MKN-45 Cell Growth in a Concentration-Dependent Manner

**Figure 1:**
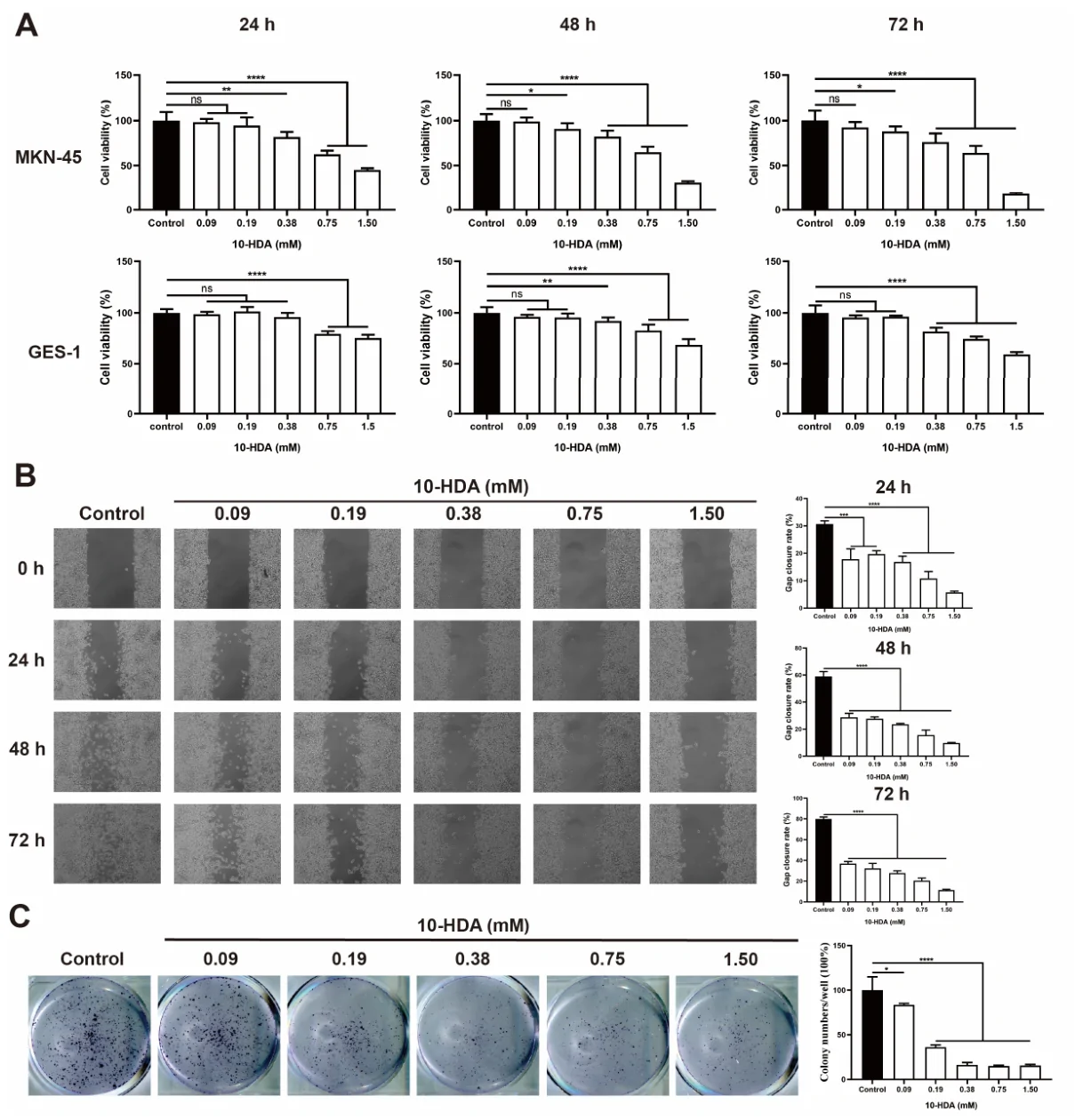
10-HDA inhibits the proliferation and colony formation of MKN-45 cells: (**A**) cell viability of MKN-45 and GES-1 cells following 10-HDA treatment for 24, 48, and 72 h; (**B**) effect of 10-HDA on MKN-45 cell scratch closure; (**C**) effect of 10-HDA on colony formation of MKN-45 cells after 15 d of treatment. Values are presented as mean ± SD. *, *p* < 0.05; **, *p* < 0.01; ***, *p* < 0.001; ****, *p* < 0.0001; ns, no statistical significance.

To investigate the anti-proliferative activity of 10-HDA on MKN-45 cells, the cells were exposed to various concentrations of 10-HDA for 24, 48, and 72 h. As shown in Figure 1A, cell viability decreased with increasing 10-HDA concentration and prolonged exposure, indicating that 10-HDA inhibited MKN-45 cell growth in a dose- and time-dependent manner. 10-HDA exhibited IC_50_ values of 1.24 ± 0.15 mM, 0.96 ± 0.08 mM, and 0.42 ± 0.06 mM following 24, 48, and 72 h of incubation, respectively. In the meantime, 10-HDA showed lower cytotoxicity against GES-1 cells. After incubation with 1.5 mM 10-HDA for 24, 48, and 72 h, MKN-45 cell viability decreased to 45.18%, 30.91%, and 17.96%, respectively, whereas GES-1 cell viability remained at 75.07%, 65.48%, and 59.08% (Figure 1A). 10-HDA did not achieve the IC_50_ concentration for GES-1 cells, even at the highest test concentration (1.5 mM). Moreover, in the scratch wound assay, 10-HDA treatment significantly delayed gap closure in the MKN-45 cell monolayers compared with the untreated control (Figure 1B). This inhibitory effect was also dependent on both concentration and incubation time. Given the 72 h duration of this assay, such a delayed gap closure is likely attributable to the anti-proliferative effect of 10-HDA. Colony formation assays further revealed that exposure to 10-HDA at concentrations ranging from 0.18 to 1.50 mM reduced colony numbers by 63.8–85.2% compared with the control group (Figure 1C). These results indicated that 10-HDA suppressed the malignant phenotypes of MKN-45 cells, including proliferation and colony formation.

### 3.2. The Effect of 10-HDA on Apoptosis

**Figure 2:**
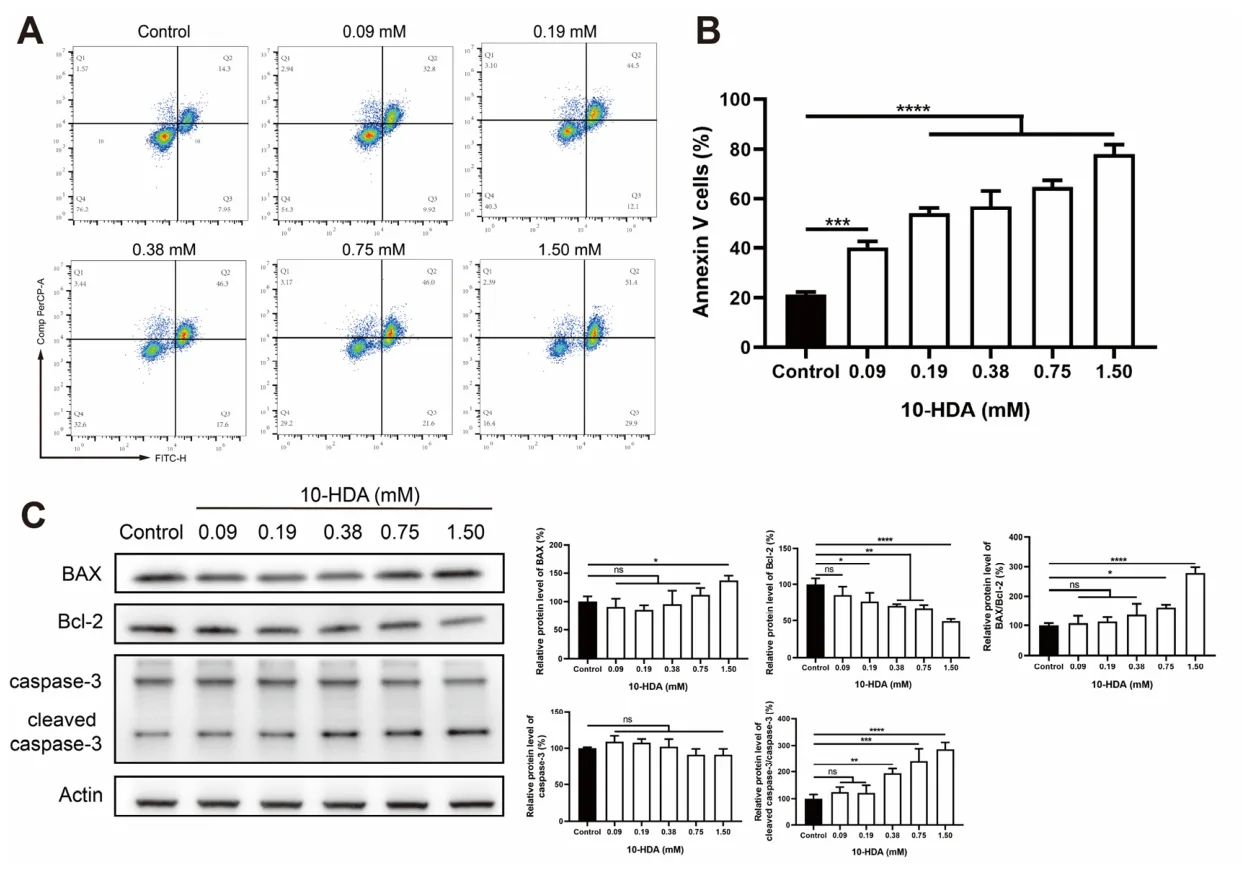
Apoptotic effect of 10-HDA on MKN-45 cells following 48 h of exposure: (**A**) flow cytometry dot plots of Annexin V-FITC/7-AAD staining; (**B**) apoptosis rates expressed as the proportion of Annexin V-positive cells; (**C**) Western blotting analysis of BAX, Bcl-2, caspase-3, and cleaved caspase-3 protein expression, and the calculated cleaved caspase-3/caspase-3 and Bcl-2/BAX ratios in MKN-45 cells treated with 10-HDA or control. Values are presented as mean ± SD. *, *p* < 0.05; **, *p* < 0.01; ***, *p* < 0.001; ****, *p* < 0.0001; ns, no statistical significance.

To further characterize the inhibitory activity of 10-HDA in MKN-45 cells, apoptosis was evaluated by flow cytometry. Using the Annexin V-FITC/7-AAD kit, cells were gated into four populations: late apoptotic (Q2, Annexin V+/7-AAD+), necrotic (Q1, Annexin V-/7-AAD+), early apoptotic (Q3, Annexin V+/7-AAD-), and live (Q4, Annexin V-/7-AAD-) cells (Figure 2A). As shown in Figure 2B, treatment with 10-HDA markedly increased the proportion of apoptotic cells in a concentration-dependent manner after 48 h of exposure. The apoptotic rate increased from 21.3% in the control group to 78.1% in the 1.50 mM 10-HDA-treated group. These results indicate that 10-HDA effectively induces apoptosis in MKN-45 cells in a dose-dependent manner. To further elucidate the molecular mechanism associated with apoptosis induction, the expression of key apoptosis-related proteins was examined by Western blotting. As shown in Figure 2C, 10-HDA significantly reduced the expression of the anti-apoptotic protein Bcl-2, whereas the level of pro-apoptotic protein BAX was markedly upregulated. Concurrently, caspase-3 activation was enhanced, as demonstrated by a significant increase in the cleaved caspase-3/caspase-3 ratio. This suggests that 10-HDA promoted apoptosis in the MKN-45 cells through modulation of apoptosis-associated proteins.

### 3.3. Transcriptome Analysis to Explore the Molecular Mechanisms of 10-HDA in MKN-45 Cells

To further elucidate the molecular mechanism underlying the effects of 10-HDA on MKN-45 cells, transcriptome sequencing was performed. This high-throughput approach enables comprehensive analysis of the composition, structure, and function of cellular transcripts under defined conditions. MKN-45 cells were treated with vehicle (control) or 1.50 mM 10-HDA for 48 h prior to sequencing (three biological replicates per treatment, six libraries total). A total of 279,788,244 raw reads were produced on the NovaSeq X Plus platform. After quality filtering, the per-sample percentage of retained clean reads, mean Q30 score, and average GC content were above 95.90%, 97.38%, and 59.17%, respectively. These values confirm the high quality and reliability of the sequencing data for subsequent analysis. The raw sequencing data in this study have been deposited in the NCBI Sequence Read Archive under accession number PRJNA1470111.

**Figure 3:**
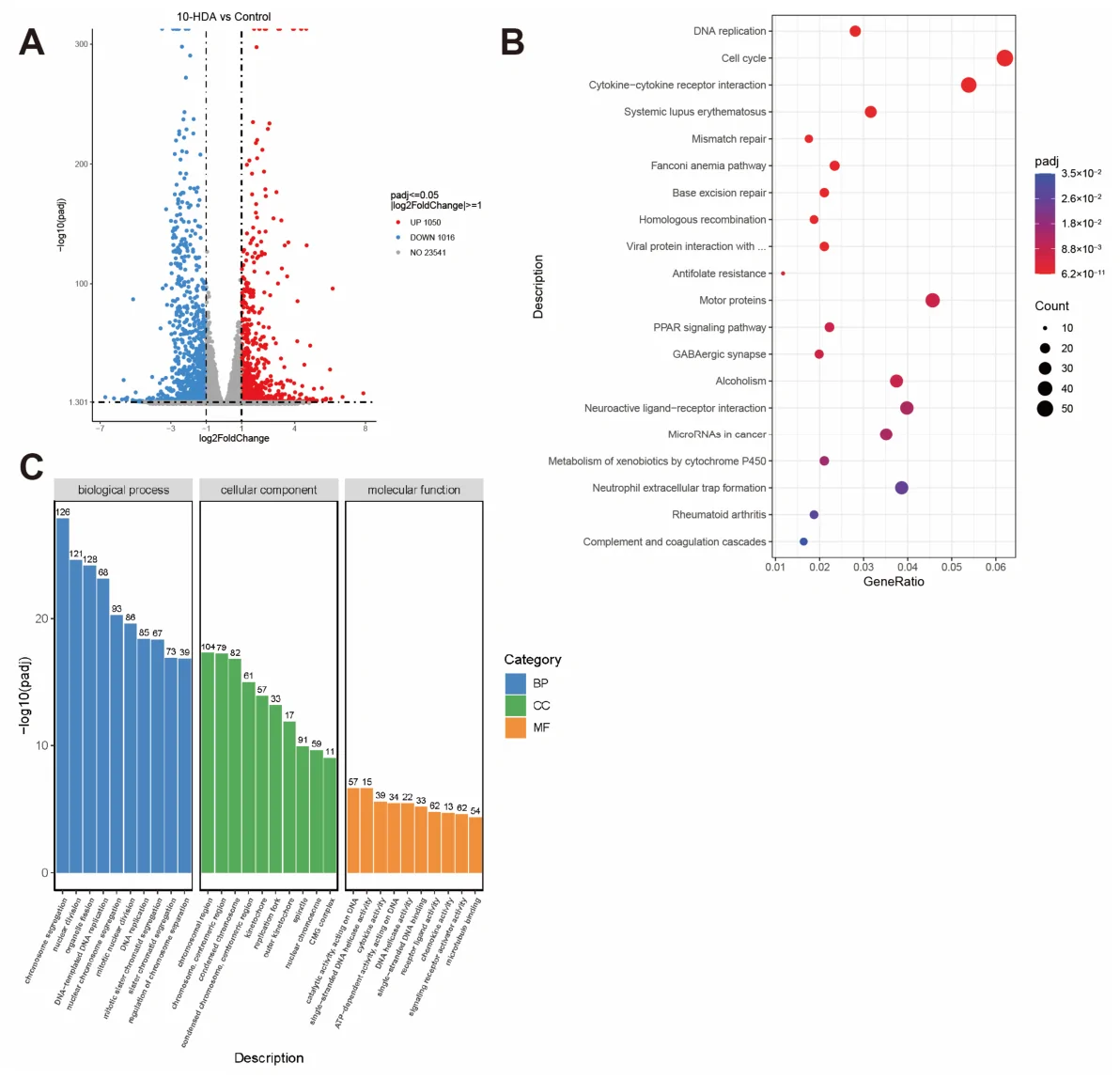
Transcriptome analysis of MKN-45 cells: (**A**) Volcano plots of DEGs, where red denotes upregulated genes and green denotes downregulated genes meeting the thresholds of FDR < 0.05 and |log2 (fold change)| ≥ 1. (**B**) Top 20 enriched KEGG pathways of DEGs, plotted by rich factor (x-axis). The size of each point represents the number of enriched genes, and its color indicates the adjusted *p*-value. (**C**) GO enrichment analysis showing top terms in molecular function (MF), cellular component (CC), and biological process (BP).

DEGs between the control and 10-HDA-treated group were identified using the criteria |log2 (fold change)| ≥ 1 and FDR < 0.05. A total of 2066 DEGs were identified, comprising 1050 upregulated genes and 1016 downregulated genes in 10-HDA-treated cells relative to the control cells (Figure 3A and Table S2). KEGG pathway enrichment analysis showed that DEGs were significantly enriched in DNA replication, cell cycle, and cytokine–cytokine receptor interaction (Figure 3B and Table S3). GO enrichment analysis revealed that the DEGs were enriched in several functional categories related to biological processes, including chromosome segregation, nuclear division, organelle fission, DNA-templated DNA replication, and nuclear chromosome segregation (Figure 3C and Table S4). These findings suggest that the anti-gastric-cancer effects of 10-HDA might be mediated through suppression of cell proliferation and replication.

### 3.4. 10-HDA Inhibits Cell Growth by Arresting the Cell Cycle

Based on the KEGG pathway and GO analyses, multiple proliferation-related genes were significantly downregulated, including proliferating cell nuclear antigen (*PCNA*), minichromosome maintenance complex component 7 (*MCM7*), cyclin A2 (*CCNA2*), cyclin-dependent kinase 2 (*CDK2*), cyclin B1 (*CCNB1*), and replication factor C subunit 3 (*RFC3*). Notably, the suppression of these genes was consistent with the reduced proliferative capacity observed in this study. This suggests that 10-HDA may exert anti-proliferative activity through inhibition of DNA replication and cell cycle progression.

**Figure 4:**
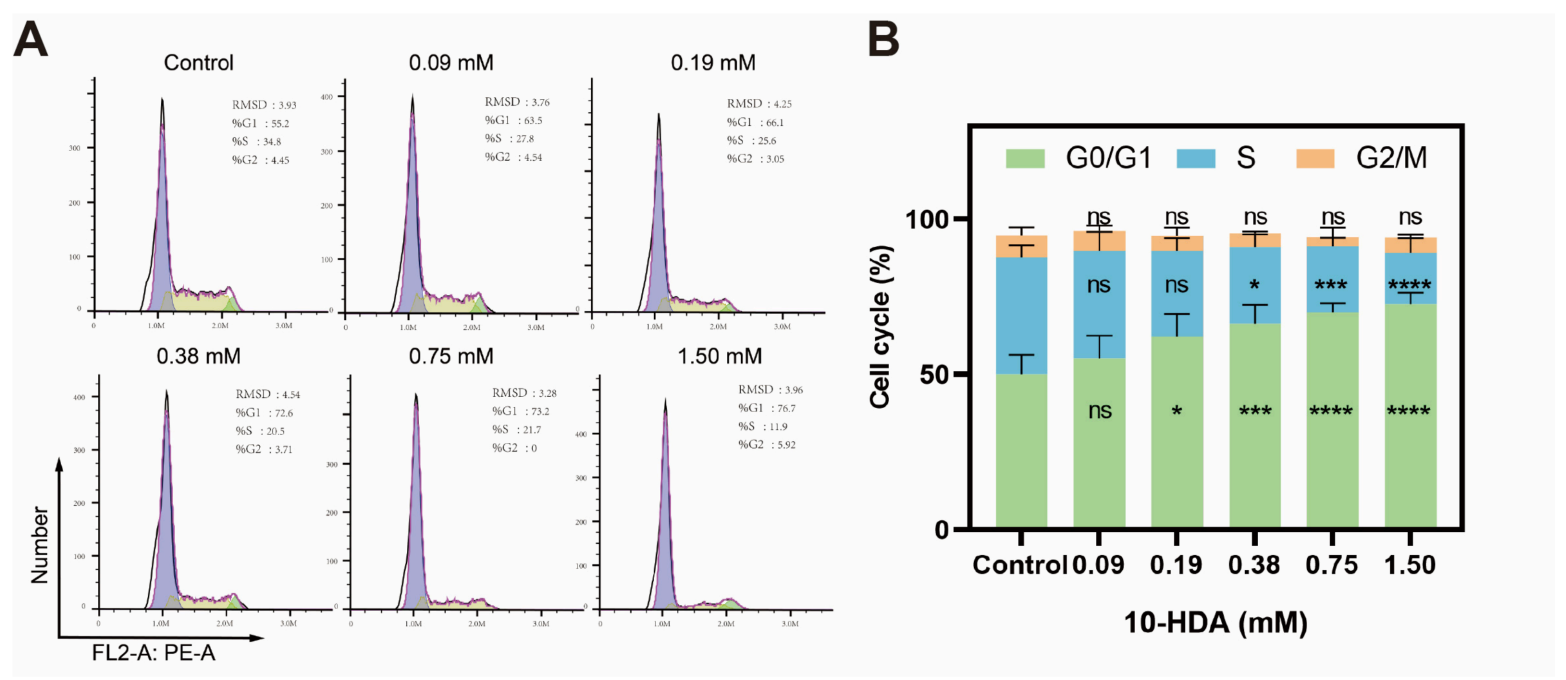
10-HDA-induced G0/G1-phase arrest in MKN-45 cells: (**A**) representative flow cytometry histograms showing cell cycle distribution following 48 h of treatment with 10-HDA (0.09–1.50 mM); (**B**) quantification analysis of cell cycle distribution. Values are presented as mean ± SD. *, *p* < 0.05; ***, *p* < 0.001; ****, *p* < 0.0001; ns, no statistical significance.

To verify this hypothesis, cell cycle distribution was first evaluated by flow cytometry. As shown in Figure 4A, treatment with 10-HDA significantly increased the proportion of cells in the G0/G1 phase, whereas the fractions of cells in the S and G2/M phases were progressively reduced. This effect became more pronounced with increasing concentrations of 10-HDA. Specifically, exposure to 1.50 mM 10-HDA increased the G0/G1-phase population to 72.6 ± 4.1%, compared with 49.9 ± 7.0% in the control group. In contrast, the proportion of cells in the S phase decreased from 37.6 ± 4.6% in untreated cells to 16.6 ± 4.9% following treatment (Figure 4B).

**Figure 5:**
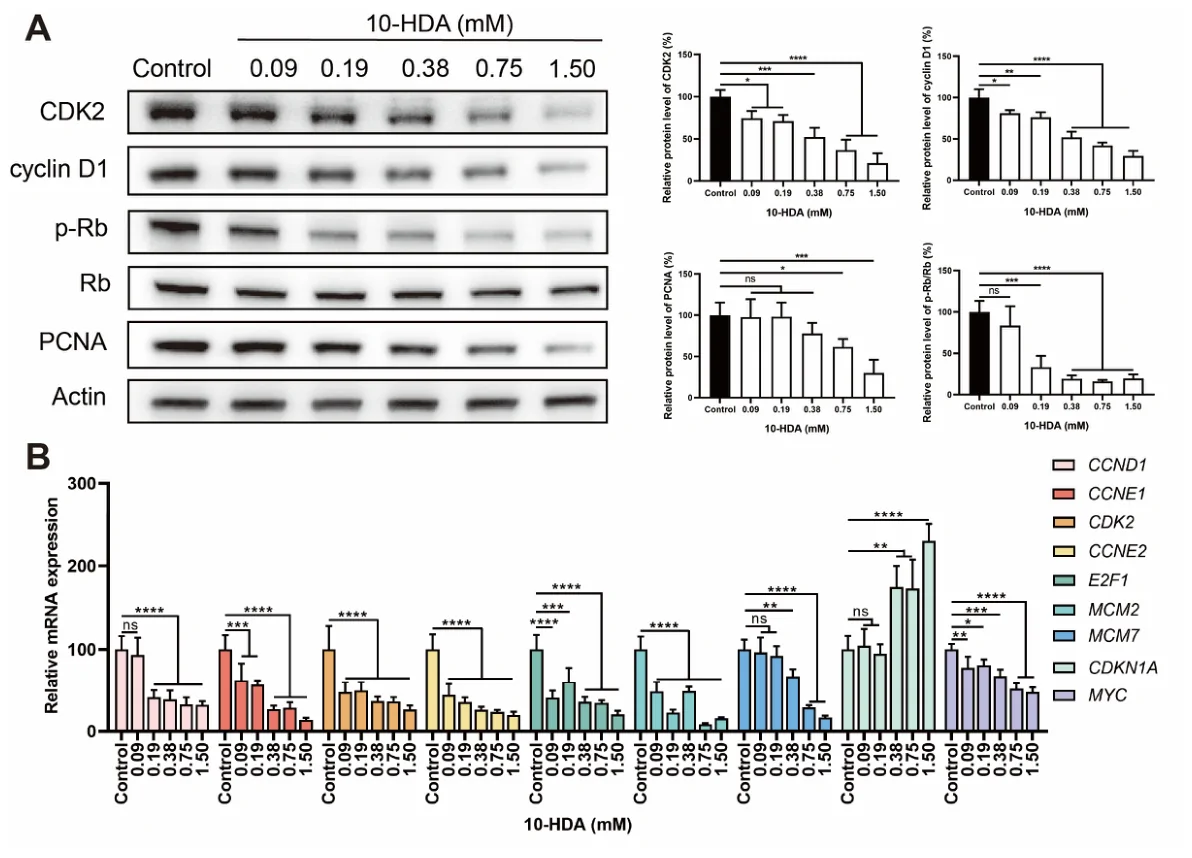
Impact of 10-HDA on the expression of key genes and proteins involved in the G0/G1- and S-phases: (**A**) Western blot analysis of CDK2, cyclin D1, p-Rb, Rb, and PCNA protein expression, together with the calculated p-Rb/Rb ratios, in MKN-45 cells treated with 10-HDA or vehicle (control); (**B**) relative mRNA expression levels of *CCND1*, *CCNE1*, *CDK2*, *CCNE2*, *E2F1*, *MCM2*, *MCM7*, *CDKN1A*, and *MYC* in MKN-45 cells treated with 10-HDA or control. Values are presented as mean ± SD. *, *p* < 0.05; **, *p* < 0.01; ***, *p* < 0.001; ****, *p* < 0.0001; ns, no statistical significance.

Given the marked alteration in cell cycle distribution, representative regulators involved in G0/G1- and S-phase progression were subsequently examined at the transcriptional and protein levels. As shown in Figure 5, RT-qPCR analysis revealed that 10-HDA significantly decreased the mRNA levels of *CDK2*, *CCNE2*, *E2F1*, *CCNE2*, *CCND1*, *MCM2*, *MCM7*, and *MYC* in MKN-45 cells, while concurrently increasing *CDKN1A* mRNA expression. At the protein level, 10-HDA markedly downregulated the protein expression of cyclin D1, CDK2, and PCNA, accompanied by a decrease in the p-Rb/Rb ratio (Figure 5). These results indicate that 10-HDA induced G0/G1-phase arrest in MKN-45 cells, thereby inhibiting cellular proliferation.

## 4. Discussion

GC is still one of the most widespread cancers worldwide, notable for its high rates of both occurrence and death [2]. Thus, the development of highly effective therapeutic agents with favorable safety profiles has become a major objective in gastric cancer research. A growing body of evidence highlights the considerable potential of functional foods and their bioactive constituents in cancer prevention and treatment [31]. A large prospective cohort study involving more than 470,000 participants reported that each 10% increase in adherence to dietary patterns enriched in functional foods was associated with a 7–12% reduction in overall cancer risk [32]. Bioactive components derived from functional foods have demonstrated significant anticancer activity across multiple malignancies. For example, curcumin, a major constituent of turmeric, has been shown to inhibit tumor development in head and neck squamous cell carcinoma, esophageal squamous cell carcinoma, and colorectal cancer [33,34,35]. Similar anticancer effects have also been reported for resveratrol from grapes, berberine from medicinal plants, quercetin from fruits and vegetables, and sulforaphane from cruciferous vegetables [36,37,38]. As a representative functional food, RJ has been reported to exhibit anticancer activity as early as 1959, and earlier studies primarily focused on its preventive effects following dietary supplementation [39,40,41]. More recently, 10-HDA, as a bioactive component of RJ, has attracted substantial research interest. Previous studies have demonstrated that 10-HDA suppresses proliferation and migratory capacity in several tumor types, including colorectal cancer, lung cancer, and lymphoma [42,43,44,45]. Nevertheless, its therapeutic potential against GC has not yet been fully characterized. In this study, the biological effects of 10-HDA on MKN-45 human gastric cancer cells were systematically evaluated, and the underlying molecular mechanisms were investigated. These findings provide further evidence supporting the anticancer activity of 10-HDA and may contribute to the development of novel therapeutic strategies for GC.

Induction of apoptosis is a core therapeutic strategy in cancer therapy [46]. This programmed cell death process is orchestrated by a diverse array of regulatory proteins, among which caspases and B-cell lymphoma 2 (Bcl-2) family members play critical roles [47,48]. BAX and Bcl-2, pro- and anti-apoptotic members of the Bcl-2 family, cooperatively regulate membrane permeabilization (MOMP). An elevated BAX/Bcl-2 ratio leads to increased MOMP, thereby triggering the release of cytochrome c from mitochondria into the cytoplasm [49]. Cytosolic cytochrome c then binds Apaf-1 and pro-caspase-9 to form the apoptosome, which activates caspase-9. In turn, active caspase-9 cleaves caspase-3 into its active form, leading to apoptosis [50]. In the current study, 10-HDA treatment upregulated BAX expression and downregulated Bcl-2 expression in MKN-45 cells, thereby increasing the BAX/Bcl-2 ratio. Concurrently, cleaved caspase-3 levels were increased, indicating effective induction of apoptosis by 10-HDA (Figure 2B). Apoptosis is typified by distinct morphological and biochemical changes, such as cell shrinkage, chromatin condensation, nuclear pyknosis, and the externalization of phosphatidylserine (PS) [51]. By labeling externalized PS with Annexin V-FITC, flow cytometry demonstrated a marked increase in the proportion of apoptotic cells, confirming that 10-HDA induced apoptosis in MKN-45 cells (Figure 2A).

Malignant tumors are characterized by uncontrolled proliferation driven by aberrant growth signaling and dysregulation of cell cycle progression. In this study, 10-HDA markedly suppressed the viability and colony-forming capacity of MKN-45 cells, indicating a strong inhibitory effect on tumor cell growth (Figure 1). Since cell cycle arrest represents a fundamental mechanism for restricting cellular proliferation, modulation of cell cycle progression has become a major strategy through which anticancer agents exert their therapeutic activity [52]. The cell cycle is composed of several distinct phases: G0/G1, S, and G2/M [53]. Flow cytometry analysis revealed that 10-HDA induced G0/G1-phase arrest in MKN-45 cells, thereby limiting progression into the S phase (Figure 4). The retinoblastoma protein (Rb) serves as a pivotal regulator of the cell cycle. In its hypophosphorylated state, Rb binds and sequesters the transcription factor E2F1 to prevent S-phase entry [54]. During early G0/G1 phase, the cyclin D1–CDK4/6 complex initiates Rb phosphorylation at specific residues. At the G1/S transition, the CDK2–cyclin E1 complex catalyzes the hyperphosphorylation of Rb at multiple sites, resulting in functional inactivation of the protein. Hyperphosphorylated Rb undergoes conformational alterations caused by the accumulation of negatively charged phosphate groups, leading to the release of E2F1 and the subsequent activation of genes required for S-phase progression [53,55,56]. In our study, the significant decrease in the p-Rb/Rb ratio and the marked suppression of *E2F1* expression were attributable to the reduced expression of cyclin D1 and CDK2, thereby arresting cells in the G0/G1 phase and preventing S-phase entry (Figure 4 and Figure 5). Simultaneously, RT-qPCR analysis revealed substantial upregulation of *CDKN1A* (Figure 5B), which encodes the p21 protein, a potent and broadly acting inhibitor of cyclin-dependent kinases [57]. Mechanistically, p21 binds to the cyclin–CDK complex through its cyclin-binding motif, which occupies a hydrophobic pocket on cyclins and competitively blocks substrate access. In addition, the kinase inhibitory domain of p21 inserts into the catalytic cleft of CDKs, inducing conformational changes that abolish kinase activity toward Rb phosphorylation [58]. This inhibitory mechanism likely contributed to the observed decrease in the p-Rb/Rb ratio [59,60]. Furthermore, the reduced expression of cyclin D1 could be attributed to the downregulation of *MYC* [61]. Given that *MYC* directly activates *CCND1* transcription, *MYC* downregulation consequently reduces *CCND1* mRNA levels, thereby leading to decreased expression of the cyclin D1 protein (Figure 5B).

Although 10-HDA exhibited cytotoxicity in GES-1 cells at high concentrations, these normal cells were significantly more tolerant than MKN-45 cells, suggesting a favorable therapeutic window for targeting malignant cells (Figure 1A). Given the G0/G1-phase cell cycle arrest induced by 10-HDA, its potential for combination therapy with conventional chemotherapeutic agents warrants further investigation. Platinum-based drugs (such as cisplatin and oxaliplatin) primarily induce apoptosis via DNA damage, which activates cell cycle checkpoints at the G1/S transition to facilitate DNA repair [62]. In this context, 10-HDA-induced G0/G1 arrest could prevent cancer cells from bypassing this checkpoint or entering the S phase for DNA repair, thereby sensitizing them to platinum-induced apoptosis and yielding a synthetic lethal effect [63]. Conversely, for antimitotic agents such as paclitaxel, the G0/G1 arrest induced by 10-HDA would likely decrease the fraction of cells entering the M phase, potentially leading to antagonism rather than synergy [64,65]. Therefore, platinum-based agents represent the most promising candidates for combination with 10-HDA to achieve enhanced anticancer efficacy.

This study demonstrated that 10-HDA suppressed the proliferation of human GC MKN-45 cells through induction of apoptosis and G0/G1-phase cell cycle arrest. The underlying mechanism appears to involve the upregulation of pro-apoptotic proteins and the downregulation of anti-apoptotic proteins, key G0/G1 proteins, and genes in the cell cycle. These findings provide a scientific and theoretical basis for the anti-gastric-cancer effects of 10-HDA, as well as support the development of novel therapeutic agents for gastric cancer. However, several limitations to this study remain: (1) The experiments were conducted exclusively in vitro using a single gastric cancer cell line; given the genetic and molecular heterogeneity of gastric cancer, it is imperative that future studies validate the broad-spectrum efficacy and molecular targets of 10-HDA across a diverse panel of gastric cancer cell lines (such as AGS and HGC-27 cells), and, ultimately, using in vivo animal models. (2) The molecular mechanisms underlying the anti-gastric-cancer effects of 10-HDA warrant further investigation, particularly its potential interactions with other signaling pathways. (3) This study focused on establishing the mechanistic baseline of 10-HDA against an untreated control; future in vitro and in vivo experiments employing standard chemotherapy drugs as positive controls are necessary to assess its relative clinical efficacy. (4) The potential synergistic effects of 10-HDA with standard chemotherapeutic agents were not evaluated. Addressing these questions in future research will be essential to further elucidate the therapeutic potential of 10-HDA.

## 5. Conclusions

In summary, this study demonstrated that 10-HDA exerted significant anticancer effects against MKN-45 human gastric cancer cells by suppressing proliferation, scratch closure, and clonogenic growth. These inhibitory effects were associated with the induction of apoptosis and G0/G1-phase cell cycle arrest, suggesting that coordinated regulation of cell survival and cell cycle progression might contribute to the anticancer activity of 10-HDA. This provides experimental evidence supporting the anticancer potential of 10-HDA and further expands the current understanding of the biological activities of the unique bioactive component of royal jelly. Further investigation is warranted to extend these results to other GC models and animal systems, to elucidate the detailed mechanisms of action, and to explore the therapeutic potential of 10-HDA in conjunction with conventional chemotherapy.

## Data Availability

The original contributions presented in this study are included in the article/Supplementary Materials. Further inquiries can be directed to the corresponding author. The original RNA sequence metadata are openly available in the NCBI Sequence Read Archive (SRA) database with the accession number PRJNA1470111.

## Supplementary Materials

The following supporting information can be downloaded at: https://www.mdpi.com/article/10.3390/nu18162670/s1. Table S1. Primers used for qPCR. Table S2. Differentially expressed genes (DEGs) in MKN-45 cells treated with 10-HDA vs. control. Table S3. KEGG enrichment of DEGs. Table S4. GO enrichment of DEGs.

## Abbreviations

The following abbreviations are used in this manuscript:

GC	Gastric cancer
RJ	Royal jelly
10-HDA	10-hydroxy-2-decenoic acid
DEGs	Differentially expressed genes
FDR	False discovery rate
GO	Gene Ontology
KEGG	Kyoto Encyclopedia of Genes and Genomes
Bcl-2	B-cell lymphoma
