# Supplementary material for: 10-HDA Suppresses the Growth of Gastric Cancer Cells Through Inducing Apoptosis and G0/G1 Cell Cycle Arrest
Source: Nutrients. 2026 Aug 15;18(16):2670. doi: 10.3390/nu18162670 (PMC13516043; doi:10.3390/nu18162670)
Supplement: Supplementary file 1 [file nutrients-18-02670-s001.zip › Supplementary Files/Table S1.pdf]

Table S1: Primers used for qPCR

Gene	Forward (5'-3')	Reverse (5'-3')
CCND1	GCTGCGAAGTGGAAACCATC	CCTCCTTCTGCACACATTTGAA
CCNE1	GCCAGCCTTGGGACAATAATG	CTTGCACGTTGAGTTTGGGT
CDK2	CCAGGAGTTACTTCTATGCCTGA	TTCATCCAGGGGAGGTACAAC
CCNE2	TCAAGACGAAGTAGCCGTTTAC	TGACATCCTGGGTAGTTTTCCTC
E2F1	CATCCCAGGAGGTCACTTCTG	GACAACAGCGGTTCTTGCTC
PCNA	CCTGCTGGGATATTAGCTCCA	CAGCGGTAGGTGTCGAAGC
MCM2	ATGGCGGAATCATCGGAATCC	GGTGAGGGCATCAGTACGC
MCM7	ACTCTCAGAAACCTACCTGGAAG	CAGCTTTTCGTAGAAATCCTCCT
CDKN1A	TGTCCGTCAGAACCCATGC	AAAGTCGAAGTTCCATCGCTC
MYC	GGCTCCTGGCAAAAGGTCA	CTGCGTAGTTGTGCTGATGT
ACTB	CACCATTGGCAATGAGCGGTTC	AGGTCTTTGCGGATGTCCACGT
